# Outcomes in patients undergoing complex cardiac repairs with cross clamp times over 300 minutes

**DOI:** 10.1186/s13019-016-0501-4

**Published:** 2016-07-12

**Authors:** Blake Shultz, Tomasz Timek, Alan T. Davis, John Heiser, Edward Murphy, Charles Willekes, Robert Hooker

**Affiliations:** Department of Thoracic and Cardiovascular Surgery, Spectrum Health, Fred and Lena Meijer Heart and Vascular Institute, 100 Michigan St. NE, 49503 Grand Rapids, MI USA; Grand Rapids Medical Education Partners, 945 Ottawa Ave NW, Grand Rapids, MI 49503 USA; Department of Surgery, Michigan State University, 15 Michigan St. NE, Grand Rapids, MI 49503 USA; 123 York Street Apt. #22V, New Haven, CT 06511 USA

**Keywords:** Adult cardiac surgery, Cross-clamp time, Mortality, Morbidity

## Abstract

**Background:**

Long cross clamp times have been associated with poor clinical outcomes, yet some patients require extremely long ischemic times to repair complex surgical problems. The purpose of this study was to examine short and mid-term survival and to identify risk factors for mortality and morbidity in patients with cross clamp times greater than or equal to 300 min.

**Methods:**

Review of our institution’s Society of Thoracic Surgeons database identified 202 patients who underwent surgical procedures necessitating aortic cross clamp times 300 min or greater between 2001 and 2012. Short-term (30-day) clinical outcomes were derived from this database and survival was assessed utilizing the Social Security Death Index. Univariate and multivariate analyses were used to determine the relationship between independent variables and mortality and postoperative outcomes.

**Results:**

The average age of the patients was 69.5 ± 10.6 (mean ± standard deviation) years and the mean ejection fraction was 52 ± 12 %. 70.3 % of patients were male. Mean cross clamp time was 346 ± 45 min, and total bypass time was 421 ± 70 min. Thirty-day mortality was 12.4 %. The incidence of bleeding and stroke were 6.4 % and 4.0 % respectively. Prolonged ventilation occurred in 26.7 % of patients, and incidence of renal failure was 10.4 %. One, three, five, and seven year survival of the patients who survived the first 30 days post-surgery was 91.9 %, 83.2 %, 75.6 % and 65.7 % respectively. Proportional hazards analysis determined that the statistically significant hazard ratios for mid-term mortality for female gender, age, and prolonged postoperative ventilation were 2.11, 1.04 and 2.72, respectively (*p <* 0.05 for each).

**Conclusions:**

Cardiac procedures requiring extremely long ischemic times have significant early mortality and morbidity. However, mid-term survival in the patients who survive is good. Decision-making regarding operability in complex cases should allow for long ischemic times.

## Background

Clinical studies have shown that aortic cross-clamp (XCL) time and cardiopulmonary bypass time (CPB) during cardiac surgery are independent predictors of mortality and morbidity in postoperative patients [1, 2, 4]. Increasing XCL times are associated with low cardiac output, prolonged ventilation, renal compromise, and neurological deficit immediately following surgery [1–5]. With the various adverse effects associated with long XCL times, mid-term survival after complex surgeries has historically had a poor prognosis. A study by Al-Sarraf et al. showed that in-hospital mortality was significantly higher for patients with XCL times greater than 90 min than for patients with XCL times >60 ≤ 90 min [1]. Furthermore, the in-hospital mortality within the latter group was significantly higher than that of patients with XCL times ≤ 60 min.

There are no large reports in the literature regarding the effects of XCL times greater than 300 min and few studies that examine mid-term survival as a function of XCL time. A large study by Doenst et al. examined data from 28,684 patients but excluded data from patients with XCL times over 120 min [4]*.* A study by Nissinen et al. examined 30-day mortality as a function of XCL time, but only included data from 30 patients with XCL times greater than 300 min [2]. Therefore, our study retrospectively examined clinical outcomes in a cohort of patients undergoing complex cardiac procedures with cross clamp times exceeding 300 min.

## Methods

### Data source

This study includes data collected from an analysis of our institution’s prospectively collected Society of Thoracic Surgeons (STS) database. All patients with primary cross clamp times over 300 min within the last 14 years were reviewed. Patients excluded included those who required a second cross clamp, any patients that had a ventricular assist device inserted and those with additional ischemic time for further repair. A total of 202 patients were identified who underwent complex surgical procedures necessitating single aortic cross clamp time 300 min or greater between 2001 and 2012. 113 patients were seen from 2001–2006 and 89 patients from 2007–2012. Our practice operates within a large, tertiary institution and saw approximately 900 patients per year until 2010 and 1,000 patients per year thereafter.

Pre-, intra-, and postoperative variables were gathered on all patients. Operative notes were obtained for data regarding the type of surgery. Short-term (30-day) clinical outcomes were derived from our STS database. Mid-term survival data (<14 years) of patients who survived the initial surgery was derived from the Social Security Death Index [23].

### Cardiopulmonary bypass

All surgeries were performed using generalized, standard anesthesia. Following a median sternotomy, a heparin dose of 350 IU/kg was given to maintain an activated clotting time of 400–600 s. Cannulation was accomplished as per the normal institutional protocol. A membrane oxygenator and roller pump system was used along with a heparin coated tubing set. 10,000 IU heparin and 1,400 mL Hartmann’s solution was used to prime the extracorporeal circuit. On average, the patients were cooled to 32 °C. Myocardial protection was accomplished with antegrade and retrograde cold blood cardioplegia in a 4:1 solution. Cardioplegia was delivered every 15 min and titrated to maintain distal septal temperature of less than 10° Celsius. A warm retrograde shot was given before clamp removal. Surgeries were performed with one aortic cross clamp. Both cardiotomy suction and cell saver were used, with cell saver used primarily for blood shed into the pericardium. Milrinone and levophed were used in the majority of patients to wean from bypass.

#### Statistical Analysis

Categorical data are expressed as percentages and quantitative variables are reported as the mean ± standard deviation (SD). The χ^2^ test and the Fisher’s Exact Test were used for the univariate analyses of categorical data and the unpaired t-test for was used for the univariate analysis of continuous data. A Kaplan-Meier survival curve was constructed from patient mortality data for those patients surviving more than 30 days postoperatively. Two logistic regression analyses were performed, using the 30-day outcomes of either mortality or complications as the dependent variables. For these analyses, independent variables tested included patient age, gender, perfusion time, cross clamp time, surgery type (whether or not the procedure included a coronary artery bypass graft (CABG)), and era (surgery performed from 2001–2006 vs. 2007–2012).

Univariate and multivariate analyses with respect to complications included eight 30-day postoperative complications. These complications are stroke, renal failure, perioperative myocardial infarction (MI), deep sternal infection, ventilation time > 48 h, atrial fibrillation, bleeding requiring reoperation and pneumonia. Stroke was defined as a confirmed neurological deficit of abrupt onset that does not resolve in 24 h. Renal failure was defined as an increase in creatinine 3.0 times over baseline, or as any requirement for dialysis. Perioperative MI was defined as new Q waves on ECG or troponin levels over 30. Deep sternal infection was defined as all of the following: (i.) wound opened with incision and drainage or any re-exploration, (ii.) positive wound cultures and (iii.) treatment with antibiotics beyond perioperative prophylaxis. Ventilation time > 48 was defined as any prolonged postoperative mechanical ventilation over 48 h. Finally, pneumonia was defined as the presence of lung infiltrate and pathogenic sputum culture, and placement on antibiotics for pneumonia.

A Cox proportional hazards analysis was performed, using mid-term mortality (occurring more than 30 days and < 14 years following the surgical procedure) as the dependent variable. The independent variables used included patient age, gender, perfusion time, cross clamp time, surgery type, era, and ventilation time >48 h. Significance was assessed at *p <* 0.05. IBM SPSS Statistics version 22.0 (Armonk, NY, USA) was used to perform the statistical analyses.

## Results

Preoperative clinical characteristics and patient demographics are summarized in Table 1. Patients ranged in age from 35 to 89 years. The majority of the patients were males, and less than 5 % of the cases were emergent. Only 10.4 % of the patients were operated on within 21 days of myocardial infarction. Over three-quarters of the patients were New York Heart Association (NYHA) class III and IV (75.3 %).

**Table 1:** Preoperative characteristics (*n* = 202)

Variable	No. (%)
Patient Age (y)^a^	69.5 ± 10.6
Gender (males, %)	142 (70.3 %)
Preoperative status	
Elective	138 (68.3 %)
Urgent	55 (27.2 %)
Emergent/salvage	9 (4.5 %)
Incidence	
Reoperation	32 (15.8 %)
First operation	
IABP - Preoperative	170 (84.2 %)
6 (3.0 %)	
Smoker	91 (45.0 %)
Angina type	
Unstable	15 (7.4 %)
Stable	65 (32.2 %)
Time from MI to surgery	
1–7 days	10 (5.0 %)
8–21 days	11 (5.4 %)
> 21 days	43 (21.3 %)
NYHA class	
≤ 2	41 (24.7 %)
3 and 4	125 (75.3 %)
Diabetes	64 (31.7 %)
Ejection fraction ≤50 %	77 (39.5 %)
Ejection fraction (%)^a^	52.0 ± 12.0

*IABP* intra-aortic balloon pump, *MI* myocardial infarction, *NYHA* New York Heart Association

^a^Mean ± SD

Table 2 presents operative data for the patient group. The majority of patients (77.3 %) underwent valve repair or replacement procedures with CABG. Mitral valve repair involved 59.5 % of the surgeries, 72.4 % involved aortic valve replacement and 16.4 % involved tricuspid repair. There were two isolated mitral valves in patients with severe mitral annular calcification. Additionally, 15.8 % of surgeries were reoperations. The isolated CABG procedures were all revisions with severe adhesions and patent grafts.

**Table 2:** Procedural characteristics and their distribution among patients within the sample (*n =* 202)^a^

Variable	No. (%)
Mitral	
+ other^1^	3 (1.5 %)
+ tricuspid + other^2^	3 (1.5 %)
Isolated	2 (1.0 %)
Aortic	
+ other^3^	13(6.4 %)
+ mitral	10 (5.0 %)
+ other^4^	8 (4.0 %)
+ tricuspid	1 (0.5 %)
Tricuspid + mitral + aortic + other^5^	6 (3.0 %)
CABG	
+ aortic	40 (19.8 %)
+ other^6^	21 (10.4 %)
+ mitral	13 (6.4 %)
+ other^7^	14 (6.9 %)
+ aortic + mitral + other^8^	21 (10.4 %) 17 (8.4 %)
+ mitral + tricuspid	2 (1.0 %)
+ other^9^	12 (5.9 %)
+ aortic + mitral + tricuspid + other^10^	9 (4.5 %)
Isolated	7 (3.6 %)

*CABG* coronary artery bypass graft

^*a*^“*Other”* surgical procedures include, but are not limited to: maze procedure (uni/biatrial)^1,2,4–10^, atrial reduction^1,2,4–10^, debridement of posterior mitral annular calcification^2,7–10^, intra-aortic balloon pump placement^4,5,6,8,9^, and patent foramen ovale closure^1,3,9^

Intraoperative and postoperative data are presented in Table 3. The mean CPB and XCL times were both more than five hours. The most frequent postoperative complications were: atrial fibrillation, prolonged ventilation >48 h, renal failure, and pneumonia.

**Table 3:** Intraoperative characteristics, 30-day postoperative outcomes, and their distributions among the patients (*n* = 202)

Variable	No. (%)
Intraoperative	
Procedure (% CABG)	156 (77.2 %)
IABP	12 (5.9 %)
CPB time (min)^a^	421 ± 70
XCL time (min)^a^	346 ± 45
IMA used	
Left or Right IMA	115/157 (73.2 %)
Both IMAs	1/157 (0.6 %)
Postoperative	
IABP	5 (2.5 %)
Operative mortality Perioperative MI	25 (12.4 %) 4 (2.0 %)
Postoperative ventilation	
≤ 48 h	148 (73.3 %)
> 48 h	54 (26.7 %)
Postoperative ventilation (h)^b^	13.0 (6.0–66.9)
Deep sternal infection	3 (1.5 %)
Stroke	8 (4.0 %)
Atrial fibrillation	53 (26.2 %)
Pneumonia	17 (8.4 %)
Renal failure	21 (10.4 %)
Bleeding requiring reoperation	13 (6.4 %)
Intensive care unit readmission	8 (4.0 %)
Total intensive care unit stay (h) ^b^	96.0 (43.0–212.3)
Total hospital stay (days) ^b^	11.0 (7.0–20.5)

*IABP* intra-aortic balloon pump, *CPB* cardiopulmonary bypass, *XCL* cross-clamp time, *IMA* inferior mesenteric artery

^a^Mean ± SD

^#^Median (interquartile range)

The results of univariate analyses with respect to patient death are shown in Table 4. The mortalities are presented as total mortalities (“all deaths”), mortalities within 30 days (“operative deaths”), and mortalities after 30 days (mid-term deaths). Female gender, and increased age, perfusion time and cross clamp time, were significantly associated with increased overall death. Perfusion time, cross clamp time, era, ventilation > 48 h, total ventilation time, total Intensive Care Unit (ICU) stay and length of hospital stay were significantly associated with increased mid-term death, while female gender and perfusion time were significantly associated with operative deaths.

**Table 4:** Univariate analysis of pre-, intra-, and postoperative variables with respect to patient *mortality*

Variable	All Deaths p-value (*n =* 72)	Mid-term Deaths^a^ *p*-value (*n =* 47)	Operative Deaths *p*-value (*n =* 25)
Era^b^	0.162	0.024	0.393
Preoperative			
Gender	0.001	0.066	0.009
Status^c^	0.186	0.691	0.157
Left main discharge >50	0.071	0.313	0.183
Patient Age	0.015	0.066	0.322
Angina	0.137	0.0497	0.699
Diabetes	0.375	0.750	0.378
Smoker	0.898	0.344	0.161
First operation vs. reoperation	0.521	0.478	>0.999
NYHA score	0.078	0.248	0.210
Intraoperative			
Surgery type^d^	0.890	0.283	0.240
Perioperative MI	0.130	0.232	0.413
Perfusion time	<0.001	0.018	0.045
Cross clamp time	0.002	0.006	0.509
Postoperative			
Deep sternal infection	-	>0.999	-
Stroke	-	0.392	-
Bleeding requiring reoperation	-	>0.999	-
Atrial fibrillation	-	>0.999	-
Pneumonia	-	>0.999	-
Ventilation >48 h	-	0.002	-
Renal failure	-	0.587	-
Ventilation time	-	0.026	-
Total ICU stay	-	0.002	-
Length of hospital stay	-	0.001	-
ICU readmission	-	>0.999	-

*NYHA* New York Heart Association, *MI* myocardial infarction, *ICU* intensive care unit

^a^All mid-term deaths were more than 30 days following the operation

^b^Surgeries from 2001–2006 were compared to those from 2007–2012; the former time period was the reference category emergent vs. elective procedures

^c^Emergent vs. elective procedures

^d^Surgeries including coronary artery bypass grafts (CABGs) were compared to those that did not

Univariate analyses with respect to complications other than mortality are shown in Table 5. Increased perfusion and cross clamp time were both associated with increased 30-day complications. Additionally, length of hospital stay, total ICU stay and ventilation > 48 h were also significantly related to increased complications.

**Table 5:** Univariate analysis of pre-, intra-, and postoperative variables with respect to *30-day postoperative complications (n = 202)*

Variable	*p*-value
Era	0.852
Preoperative	
Gender	0.248
Status	0.285
Angina	0.477
NYHA Score	0.613
Diabetes	0.450
First operation vs. reoperation	0.381
Smoker	0.877
Patient age	0.731
Perioperative	
Perfusion time	0.003
Cross clamp time	0.040
Postoperative	
Length of hospital stay	<0.001
Total ICU stay	<0.001
Ventilation >48 h	<0.001

*NYHA* New York Heart Association, *ICU* intensive care unit

Logistic regression analysis was performed using 30-day mortality or 30-day complications as the dependent variables (Table 6). Female gender was the only independent variable that was significantly predictive of mortality. Females were 2.44 times more likely to die than males. None of the independent variables were statistically significant predictors of 30-day complications.

**Table 6:** Logistic regression analysis of independent variables with respect to 30-day mortality and complications^a^

	30-day Mortality		30-day Complications	
Variable	OR (95 % CI)	*p*-value	OR (95 % CI)	*p*-value
Patient age	1.02 (0.98–1.07)	0.305	1.00 (0.97–1.03)	0.902
Gender^b^	2.44 (1.004–5.93)	0.049	1.19 (0.62–2.31)	0.599
Perfusion time	1.01 (1.00–1.01)	0.113	1.01 (1.00–1.01)	0.139
Cross clamp time	0.99 (0.98–1.01)	0.362	1.00 (0.99–1.01)	0.968
Surgery type^c^	0.64 (0.24–1.71)	0.377	0.53 (0.25–1.12)	0.097
Era^d^	1.17 (0.48–2.83)	0.725	0.90 (0.50–1.63)	0.726

*ICU* intensive care unit

^a^All mortalities for this analysis occurred perioperatively

^b^The reference category was males

^c^Surgeries including coronary artery bypass grafts (CABGs) were compared to those that did not; the reference category was surgeries involving CABG

^d^Surgeries from 2001–2006 were compared to those from 2007–2012; the former time period was the reference category

Cox proportional hazards analysis was also performed using mid-term mortality for patients who survived at least 30 days following the surgery as the dependent variable. Significant predictors of mortality included female gender, age, and ventilation > 48 h (Table 7).

**Table 7:** Cox proportional hazards regression analysis of independent variables with respect to the mid-term mortality^a^

Variable	OR (95 % CI)	*p*-value
Gender^b^	2.11 (1.13–3.94)	0.020
Perfusion time	1.00 (0.99–1.01)	0.933
Cross clamp time	1.01 (0.999–1.02)	0.081
Surgery type^c^	1.44 (0.65–3.22)	0.372
Era^d^	1.06 (0.53–2.13)	0.861
Age	1.04 (1.004–1.07)	0.027
Ventilation > 48 h	2.72 (1.46–5.08)	0.002

^*a*^All mortalities occurred more than 30 days after the surgical procedure

^b^The reference category was males

^c^Surgeries including coronary artery bypass grafts (CABGs) were compared to those that did not; the reference category was surgeries involving CABG

^d^Surgeries from 2001–2006 were compared to those from 2007–2012; the former time period was the reference category

**Fig. 1:**
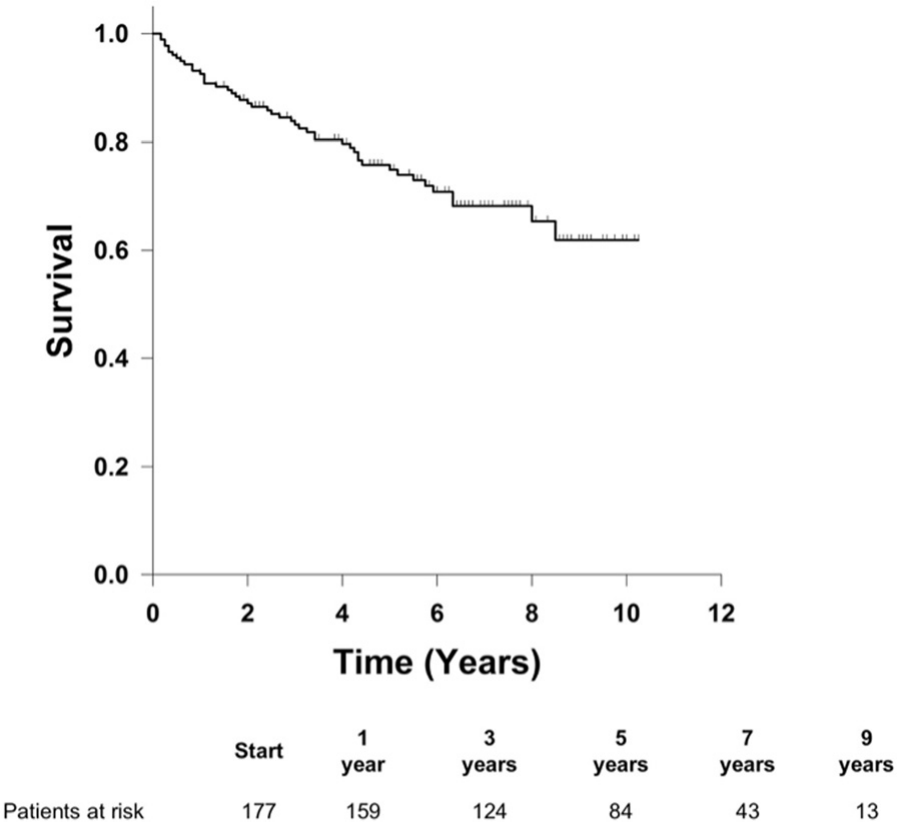
Kaplan-Meier survival curve predicting survival of patients

A Kaplan-Meier survival curve is shown in Fig. 1 for patients who survived at least 30 days following surgeries with cross clamp times greater than 300 min. One, three, five, and seven year survival was 91.9 %, 83.2 %, 75.6 %, and 65.7 % respectively.

## Discussion

Complex surgical procedures requiring long cross clamp times for complete repair are often patients’ only chance for survival and better quality of life. Decisions about operability are often difficult, as research has shown that XCL time is an independent predictor of mortality and morbidity in patients undergoing cardiac surgeries [1, 2, 4]. Our results demonstrate that while high in relation to less complex surgeries, a 12.4 % mortality in these complex, high risk patients is lower than that reported by studies in the literature addressing longer XCL times. Nissinen et al. observed a 31.5 % 30-day mortality rate in their patients with XCL times greater than 240 min [2]. Furthermore, Al-Sarraf et al. found that high risk patients (EuroScore ≥ 6) with XCL times >90 min and those with XCL times < 60 min and ≤ 90 min were respectively 4.7 and 3.1 times more likely to die than those with XCL times ≤ 60 min [1]. Doenst et al. found that XCL times greater than 30 min were associated with a steadily increasing rate of mortality [4].

Our results differ slightly from the above findings. In our cohort, perfusion time was significantly associated with all deaths, mid-term deaths and operative deaths by univariate analysis, while XCL time was associated with all deaths and mid-term deaths only. Both perfusion time and XCL time were significantly associated with 30-day complications by univariate analysis. Neither perfusion time nor XCL time was significantly associated with mortality or complications by multivariate analysis, although XCL time was associated with mid-term mortality with *p =* 0.081 under Cox proportional hazards analysis. Studies have shown that modern methods of myocardial protection have greatly reduced the mortality expected from long XCL times. Bezon et al. showed an absence of increased mortality and morbidity during prolonged cross-clamping with continuous retrograde tepid cardioplegia with systemic normothermia [7]. Furthermore, Bar-el et al. found no increase in mortality in operations requiring more than two hours of cross-clamp time with the use of antegrade administration of warm, undiluted blood and continuous retrograde infusion of tepid, undiluted blood with added potassium and magnesium [10]. Myocardial protection is extremely important and a standardized approach was utilized with cardioplegia delivered every 15 min during cross-clamping. Septal temperatures were monitored and kept under 10° Celsius. Right ventricular protection via grafts to the right side and integrated into the cardioplegia circuit is used if there is disease. Antegrade cardioplegia in the root or ostial right perfusion is used if there is no disease, with the addition of ice on the right ventricle surface.

CPB duration has been shown to be positively correlated with interleukin-6 response that is responsible for the systemic inflammatory response (SIR) associated with adverse outcomes in cardiac surgery [8]. SIR is multifactorial in cause, but evidence suggests that blood-to-artificial surface contact leads to platelet activation and results in an altered platelet shape. This altered shape activates cascades producing coagulants, complement and inflammatory cytokines like IL-6 [8]. Furthermore, studies such as that by Wan et al. showed increasing levels of Il-6, Il-8, and Il-10 with longer ischemic times [13]. Thus with extremely long cross clamp times there is a possibility of increased inflammatory mediators which can lead to increased incidence of complications through the SIR. Heparin coated circuits and the avoidance of cardiotomy suction has been used as a possible means to decrease inflammatory mediators and thus SIR.

A number of studies have reported the correlation between prolonged XCL and bypass time and renal compromise [1, 3, 6, 11]. This acute kidney injury is multifactorial and related to preoperative renal function as well as intraoperative factors such as hypoperfusion, emboli, and medications. Taniguchi et al. showed that when CPB times exceed 90 min, the chance of renal dysfunction, an independent risk factor for mortality, increased significantly [14]. In 2003 Boldt et al. measured urinary proteins excreted and found that with bypass times greater than 90 min these markers of renal injury were elevated and remained so compared to those without these times [15]. In our cohort, renal failure was classified as an increase in creatinine of 3.0 times over baseline or as any patients requiring dialysis. While renal failure was not significantly associated with mortality by univariate analysis, a relatively large number (10.4 %) of patients experienced renal failure. A review by Rosner and Okuza showed that mortality in patients requiring dialysis after cardiac surgery was uniformly high in fifteen studies, at an average of 60–70 % [24]. Therefore, optimization of preoperative renal function by delaying surgery until all effects from dye administration are gone, discontinuing nephrotoxic medications and renal consultation in these patients is crucial. Intraoperative management by avoiding hypotension and severe hemodilution can be utilized to mediate renal complications [16].

Prolonged postoperative ventilation (>48 h) was significantly associated with both 30-day and mid-term mortality in our cohort under univariate analysis. A high incidence of pneumonia, prolonged ventilation and other pulmonary complications have been shown in prior literature [1, 3, 12]. For example, Al-Sarraf et al. showed that XCL times greater than or equal to 90 min put a patient at 1.6 times the risk of pulmonary complications compared to patients with a shorter ischemic time [1]. One study suggests that this is likely a result of increased vascular resistance in the lungs, and the accumulation of anaerobic metabolic byproducts in blood flowing through the pulmonary circuit following declamping in addition to a postoperative increase in membrane permeability with subsequent pulmonary edema causing further complications [9]. It has been suggested that the decrease in arterial oxygen tension and other pulmonary imbalances due to heparin use are caused by perioperative pulmonary microemboli [17]. Leukocyte depletion filters and a reduced use of the cardiotomy suction device can both be utilized to reduce postoperative pulmonary complications [18].

A high incidence of stroke has been shown to occur with prolonged XCL [1]. A 2000 study demonstrated that longer durations of bypass were associated with increased embolic load on the brain [19]. A moderate incidence of stroke was observed in this study (4.0 %), although this is consistent with other studies in the literature [1, 5]. Although the correlation between the incidence of stroke and mortality did not reach statistical significance in our study, of the 4.0 % who suffered a stroke, 75 % (6/8) died within thirty days of the procedure, as compared to 12.4 % of the entire cohort. A cohort with a higher number of patients may demonstrate a statistically significant relationship with the incidence of postoperative stroke and mortality. Although previous research has reported a high incidence of complications such as renal failure and stroke with prolonged XCL, the correlations between these complications and XCL time in these studies was not assessed [1, 5, 11]. In the past ten years, routine preoperative CT scanning of the thoracic aorta as well as intraoperative epiaortic echocardiography and cerebral oximetry have been utilized to attempt to reduce the incidence of stroke.

Multiple studies have suggested that female gender is associated with increased mortality and morbidity [20–22]. In our cohort, female gender was significantly correlated with mid-term mortality by Cox proportional hazards analysis, and was significantly associated with 30-day mortality by logistic regression. Our results also demonstrated a 2.44 times higher 30-day mortality and a 2.11 times higher mid-term mortality in females who underwent operations requiring prolonged cross clamp times. Blankenstein et al. demonstrated double the operative mortality in women who underwent CABG surgery [20]. Abramov et al. also showed an increase in early mortality in women (2.7 %) vs. men (1.8 %) after CABG surgery [21]. There are many possible explanations for this increased operative mortality. Abramov et al. suggest that women have significantly increased incidences of preoperative risk factors such as diabetes, hypertension, peripheral vascular disease, congestive heart failure, urgent operation and angina class 3 or 4 relative to men [21]. Similarly, Tran et al. suggest that women have more complex morphology due to under-diagnosis of valve disease, and a general underestimation of the severity of cardiac illness in females [22]. Finally, Blankenstein et al. suggest that the lower BSA of females relative to males acts as a surrogate for smaller coronary vessel size, which may lead to reduced graft patency and negatively influence mid-term survival. The same study found that when mortality is risk adjusted for this higher prevalence of preoperative complications and lower BSA, females still have 22 % higher operative mortality than males [20].

Despite the above risk factors and postoperative complications, patients who survived thirty days postoperatively and were discharged from the hospital had excellent mid-term survival. One, three, five and seven year survival in our cohort was 91.9 %, 83.2 %, 75.6 % and 65.7 % respectively. The era in which the surgery took place was not significantly associated with complications or mortality by multivariate analysis.

These data demonstrate that the prognoses for complex surgeries with extended XCL times are reasonable and that it may be reasonable to offer surgery to very complex patients who may have been refused in the past. Optimization of patients prior to undergoing these complex procedures is vital for improving outcomes. Waiting 5–7 days after a catheterization or CT angiogram minimizes any potential of nephrotoxicity in elective cases. Holding anticoagulants can minimize the deleterious effect of blood transfusions, and maximizing cardiac medications such as beta-blockers and blood pressure medication may benefit patients. Surgeries involving patients with high HgA1c levels should be delayed until said levels are controlled. Finally, from a lifestyle perspective, insisting that patients stop smoking and requiring patients to be ambulatory as soon as possible (with the goal of achieving twenty minutes of continuous activity) may also enhance beneficial outcomes.

Limitations to this study include it being a retrospective review of prospective data. For mid-term data, only survival was monitored and we were not able to assess quality of life. There is also no ability to assess why cross clamp time was so long as operative notes were absent or incomplete and we were unable to discern all problems encountered, however we excluded patients who needed to have the cross clamp applied a second time to attempt to minimize this. Another possible limitation to this study is the small sample size (*n =* 202). Finally, the study is limited in its design as a single institution study over a long period of time. However, after stratifying patients by time, we found that era is not a statistically significant predictor of mortality or postoperative complications.

## Conclusion

In conclusion, there is significant but acceptable short-term mortality and morbidity in this cohort of complex patients. There is also excellent mid-term survival in patients who survive the initial operation. Female gender was significantly associated with both 30-day mortality and mid-term mortality by multivariate analysis. Patient age and prolonged postoperative ventilation were significantly associated with mid-term mortality by cox proportional hazards analysis. There are few studies that look at mid-term survival in patients undergoing these surgeries, and our findings demonstrate a need for further longitudinal studies in these patients. Future studies should focus on preoperative optimization of these patients in terms of respiratory function and renal function. Postoperative care also needs to be studied and optimized, which we believe incorporates a multidisciplinary team approach to these complex patients.

## Abbreviations

XCL, cross-clamp; CPB, cardiopulmonary bypass; STS, Society of Thoracic Surgeons; SD, standard deviation; CABG, coronary artery bypass graft; MI, myocardial infarction; NYHA, New York Heart Association; ICU, Intensive Care Unit; SIR, systemic inflammatory response; IABP, intra-aortic balloon pump; IMA, inferior mesenteric artery
